# Enhancing cross-evidence reasoning graph for document-level relation extraction

**DOI:** 10.7717/peerj-cs.2123

**Published:** 2024-06-17

**Authors:** Qiankun Pi, Jicang Lu, Taojie Zhu, Yepeng Sun, Shunhang Li, Jiaxing Guo

**Affiliations:** 1 PLA Strategic Support Force Information Engineering University, Zhengzhou, Henan, China; 2 State Key Laboratory of Mathematical Engineering and Advanced Computing, Zhengzhou, Henan, China

**Keywords:** Document-level RE, Evidence graph, Entity-level graph

## Abstract

The objective of document-level relation extraction (RE) is to identify the semantic connections that exist between named entities present within a document. However, most entities are distributed among different sentences, there is a need for inter-entity relation prediction across sentences. Existing research has focused on framing sentences throughout documents to predict relationships between entities. However, not all sentences play a substantial role in relation extraction, which inevitably introduces noisy information. Based on this phenomenon, we believe that we can extract evidence sentences in advance and use these evidence sentences to construct graphs to mine semantic information between entities. Thus, we present a document-level RE model that leverages an Enhancing Cross-evidence Reasoning Graph (ECRG) for improved performance. Specifically, we design an evidence extraction rule based on center-sentence to pre-extract higher-quality evidence. Then, this evidence is constructed into evidence graphs to mine the connections between mentions within the same evidence. In addition, we construct entity-level graphs by aggregating mentions from the same entities within the evidence graphs, aiming to capture distant interactions between entities. Experiments result on both DocRED and RE-DocRED datasets demonstrate that our model improves entity RE performance compared to existing work.

## Introduction

Relation extraction (RE) aims to automatically detect and extract connections between entities, unveiling the underlying semantic relationships within a given text. Traditional RE focuses on the sentence-level (Liu et al., 2020; Zeng et al., 2014; Alt, Gabryszak & Hennig, 2020; Peng et al., 2020), predicting relationships between entities that are present within a single sentence. However, in tasks that require cross-sentence RE, many relation instances are distributed across multiple sentences. Sentence-level RE concentrates solely on information contained within individual sentences, thereby limiting its effectiveness for extracting relations between entities across different sentences.

**Figure 1:**
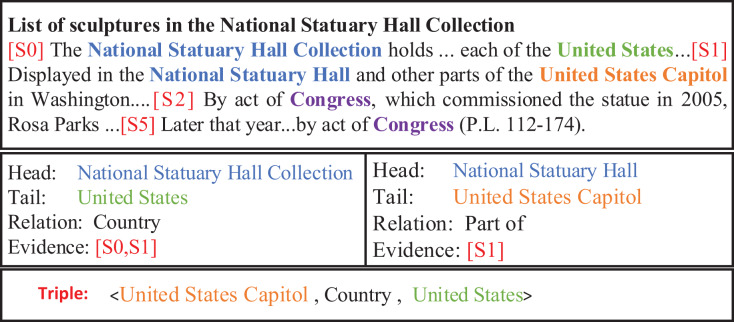
An example from the DocRED dataset.

In document-level RE, the distribution of entities is imbalanced, which requires comprehensive consideration of the context information from multiple sentences for relation prediction among cross-sentence entities. This phenomenon further complicates document-level RE. As shown in Fig. 1, it is easy to recognize the relationship between the entities (*National Statuary Hall Collection*, Country, *United States*) and (*National Statuary Hall*, Part of, *United States Capitol*) in the same sentence. However, it is not easy to directly predict the relationship between the cross-sentence entities *United States Capitol* and *United States*.

Existing studies constructed document-level graphs from various sources such as syntax trees and unstructured text to enhance cross-sentence relation reasoning. Sahu et al. (2019) introduced a graph convolutional network (GCN) featuring edge annotations at the document level, aiming to capture dependency data from both local and non-local sources. Christopoulou, Miwa & Ananiadou (2019) proposed a model that leverages an automatically inferred document-level graph to bolster reasoning about relations spanning multiple sentences. Nan et al. (2020) proposed that the model automatically induces potential document-level graph allow the model to progressively gather pertinent information for multi-stage inferential processing about inter-entity relations. However, these methods tend to introduce noise during entity information integration, which can impair the performance of the model.

Unlike their work, we work on incorporating evidence into the graph, which not only reduces the complexity of composing the entire document, but also reduces the noise of the composition more, allowing the model to focus only on the context related to entity relationship extraction. Therefore, we propose an enhancing cross-evidence reasoning graph model for document-level RE. Specifically, we propose an extraction rule for evidence based on center-sentence. Based on the revised rule, we pre-identify a set of evidence corresponding to mention pairs that might be related and integrate them into a pseudo-document. The model can focus on the sentences related to a given entity. Next, we establish an evidence graph on this, in which we can learn the relevant contextual information in the evidence according to the pseudo-document. Then, we merge mentions of the same entity in the evidence graph to construct an entity-level graph and utilize it to capture path information between entities. Finally, we concatenate the representations of the head and tail entities with the information from the inference path between them to predict the relation.

The principal contributions of our work are as follows:

- We design a center-sentence extraction rule to better capture diverse semantic expressions in real document contexts and obtain higher quality evidence.
- We propose a novel model that incorporates evidence into a cross-evidence reasoning graph. This model identifies pairs of mentions with same evidence from different sets and leverages cross-evidence interaction to enhance the model’s understanding of the relationship between entities through information transfer and sharing.
- Our experimental results on both DocRED and RE-DocRED datasets further demonstrate that our model improves cross-sentence entity RE performance, validating the importance of introducing cross-evidence reasoning graph.

## Related work

In this section, we will mainly introduce the related work of evidence and graph-based document-level relation extraction. Specifically, first, we discuss related work on document-level relation extraction based on evidence, and summarize the current work on document-level relation extraction using evidence. Then, we summarize the research on graph-based document-level relation extraction, and summarize a variety of cutting-edge methods for document-level relation extraction using graph neural networks.

### Document-level RE based on evidence

In document-level RE tasks, a significant portion of a document may be irrelevant to RE, using the whole document for RE will add the sophistication of the model. By introducing evidence, the modeling task can be simplified, making the model more focused on processing relations within local contexts.

Xie et al. (2021) introduced a evidence-enhancing perceptual model by designing an effective path search strategy prior to evidence extraction, focusing on more important sentences. Huang et al. (2021a) proposed a new entity and evidence-guided RE framework that focuses the model on the relevant regions of the document, helping the model in directing its attention towards the sections of the document that hold greater relevance to the head entity. Huang et al. (2021b) proposed three heuristic rules to extract higher-quality evidence, selecting information-rich path sets from the whole document and reducing the noise interference of irrelevant sentences. Based on Huang et al. (2021b), Zhu et al. (2023) added heuristic rules combining neighbor sentences and bridge entities on top of the original rules to enhance entity representation. Both Huang et al. (2021b) and Zhu et al. (2023) have well-designed evidence extraction rules.

However, their default rule treats sentences containing head and tail entities as evidence, which is imperfect. The difference is that the evidence extraction rules we construct are more from the perspective of realistic text. We believe that the first sentence in the document is usually the central sentence of the article, and the sentences containing more entities are often the central sentence of the article. Therefore, when other rules are not suitable, we consider setting such rules to extract evidence sentences.

### Document-level RE based on graph

In the document, there are long-distance dependency relations between certain entities, which may span across the entire document. Graph-based modeling approaches have the capability to handle such long-distance dependencies, effectively integrating contextual information.

In the existing work, the graph structure is used to represent the entities in the document and the relationships between them, and the graph has great advantages in capturing the complex relationships between the entities. Christopoulou, Miwa & Ananiadou (2019) proposed using different types of nodes and edges to create document diagrams. The inference mechanism at the edge of the graph makes it possible to use internal multi-instance learning to learn relationships within and between sentences. Sun et al. (2022) proposed to make further use of global topological information by measuring the importance of nodes, combining these non-local relations into GCN to aggregate related information. In addition, to simulate semantic and syntactic interactions in documents, a new strategy is designed to construct document-level heterogeneous graphs with different types of edges. Zeng et al. (2020) constructed a network for graph aggregation and reasoning that involves creating both mention-level and entity-level graphs, alongside a novel method of path-based reasoning to deduce the interrelations among entities. Ding et al. (2023) constructed a collaborative local-global reasoning network, designed to effectively predict relationships between entities by integrating information from both local and global sources.

However, these graph-based approaches mentioned above only consider modeling the entities themselves and cannot effectively use the semantic cues within sentences to predict the relationships among pairs of entities. Therefore, Liu et al. (2023) developed a document-level graph to emulate the semantic interplay of mentions and sentences in a document. However, the construction of the graph incorporates sentence information unrelated to RE, leading to the computation of unnecessary sentences and the introduction of interference from noise.

The difference is that we propose to integrate evidence into the graph, take the set of evidence as an important basis for predicting entity relationships, combine and connect the mention pairs with the same evidence in the set of evidence, effectively capture the correlation between different evidence, and reduce the recurrence of the same information in the model. The potential semantic information between the mentioned pairs under the same evidence is also mined.

## Problem description and analysis

Given a document D consisting of N sentences $\left\{ {{s_n}} \right\}_{n = 1}^N$, each containing a collection of entities $E = \{ {e_m}\} _{m = 1}^M$. Our task is to find the connection between entity ${e_i}$ and entity ${e_j}$. In document-level RE tasks, entities can be distributed across different sentences, necessitating the resolution of predicting relations between entities in disparate sentences. In this article, we aim to find higher-quality evidence by constructing heuristic evidence rules, constructing evidence graph using evidence to mine semantic information between cross-sentence entities. By constructing entity-level graphs by merging identical entity mentions in the evidence graph to reason about entity pairs in order to better predict their relations.

## Methodology

In this section, we describe our proposed enhancing cross-evidence reasoning graph for document-level RE. The model is composed of four primary sections. The first component is the evidence extraction module, which aims to extract higher quality evidence from documents. The second is the encoder module, which converts the words within the document into the corresponding vector representation. The third part is the graph construct module, which constructs the evidence graph by extracting the evidence obtained in evidence extraction, mining the entity information with the same evidence. Then combines the references of the same entity in the evidence graph to construct an entity-level graph, so as to better reason the interaction between entities. The last part is the relation classifier, which is used to represent the target entity pair, classify and predict the relationship between entities.

### Evidence extraction

**Figure 2:**
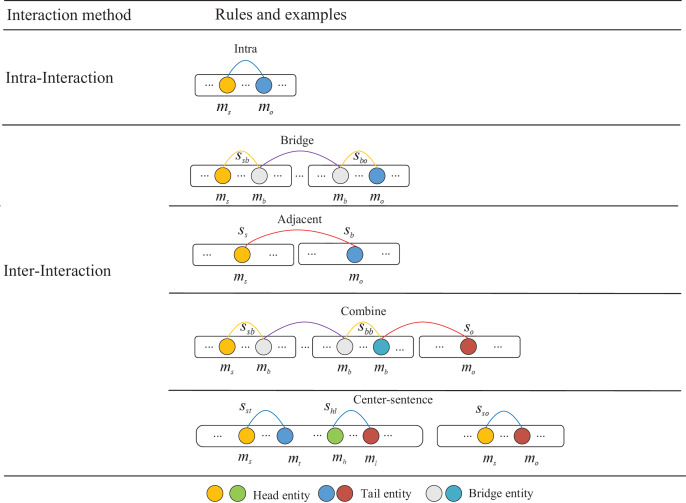
Description of interaction types of evidence extraction rules. Different colored circles represent different types of entities.

In the process of extracting evidence, it is a complex task to establish the entity association among the mixed information of multiple sentences, which usually requires the analysis of cross-sentence relationships, and the relationships between sentences are relatively chaotic, so it is difficult to explicitly determine the relationship between entity pairs between sentences. To utilize higher-quality evidence for mining semantic interactions among entities, we enhance default rules based on Zhu et al. (2023), taking into account the semantic information of complex real-world documents. We employ the center-sentence rule to get more precise collection of evidence. The detailed extraction rules and the sequential order of the evidence are shown in Fig. 2.

**Figure 3:**
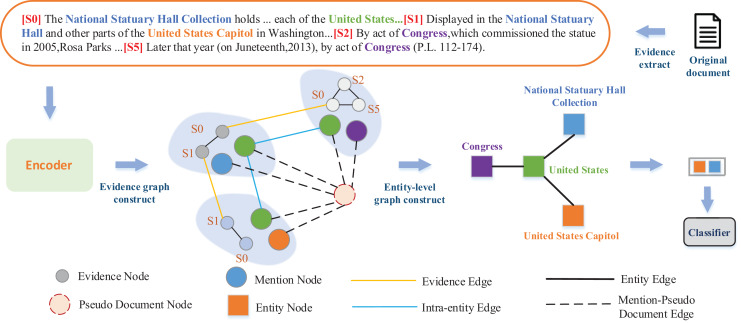
Architecture of our proposed model. The irregularly shaped light shaded sections represent the concept of a subgraph, and the shaded sections for each region represent a subgraph composed of mention pairs and associated evidence.

- Intra-sentence rule: For a pair of entities in the same sentence, if there is a relationship between the two entities, we can assume that the sentence is likely to contain evidence of a relationship between the two entities. Therefore, we use this sentence as an evidence between two entities.
- Bridge entity rule: Some entities in the article may not be directly linked, but a bridge can be built in the middle through other entities to connect the two entities. By analyzing the relationship between bridge entity, origin entity and target entity. We can further infer the relationship between the origin entity and the target entity.
- Adjacent-sentence rule: The phenomenon where entities in some sentences in the document cannot be connected through a bridge entity, but often have some close connection with their neighboring sentences, leads to the proposal of the neighboring sentence rule. This rule considers the neighboring sentence as evidence for the relationship between the two entities. Determine the relationship between the starting and target entities by searching for adjacent sentences. For example, the evidence [S0,S1] in Fig. 3 that refers to the *United States* and the *National Statuary Hall Collection* are found by the rule of the adjacent-sentence.
- Combine rule: For some complex entity interactions, it is often necessary to infer the relationship between the two entities by combining the bridge entity rules and the neighboring sentence rules. First, we use adjacent-sentence rule to capture relationships between entities across sentences, and then we use bridge entity rule to infer associations between two entities. By combining the two rules, more complex relationships between entities are captured. For example, the evidence [S0,S2,S5] in Fig. 3, that refers to the *United States* and the *Congress* are found by the rule of the combine.
- Center-sentence rule: After considering the actual context of the text and conducting statistical experiments, we find that the first sentence in the text is usually the center-sentence that contains most of the entities and relationships. In addition, sentences that contain most pairs of entities also follow the center-sentence rule, that is, they usually appear in the same sentence and have the same evidence. Therefore, in cases where none of the above rules apply, we use the center-sentence rule. This rule fully considers the actual context information and can significantly improve the recall rate of evidence extraction. For example, the evidence [S0,S1] in Fig. 3 that refers to the *United States* and the *United States Capitol* are found by the rule of the center-sentence.

To evaluate the performance of our evidence rule in extracting evidence. We use Precision and Recall to evaluate the quality of the extracted evidence sentences, where Precision represents the proportion of the evidence correctly extracted by the model to the real evidence, and Recall represents the proportion of the evidence correctly extracted by the model to all the evidence extracted. Simply considering one indicator is one-sided, therefore we use Evi-F1 to combine the Precision and Recall, so as to better judge the quality of evidence.

We present the statistics of our evidence extraction results on the DocRED development set in Table 1. In addition, HESRE (Zhu et al., 2023) and DRE-EA-path (Xu et al., 2022) respectively use their own rules to extract evidence sentences. HESRE uses its own heuristic rules to extract evidence, while DRE-EA-PATH uses reinforcement learning to select evidence. As can be seen from the table, the Precision and Evi-F1 value of our evidence rule are 1.03% and 0.23% higher than that of HESRE, and 5.6% and 3.41% higher than that of DRE-EA-path, respectively. This improvement indicates that the center-sentence rule proposed by us can be effectively applied to the actual context information of the document. It further shows the advantage of this rule in the prediction of inter-sentence entity relationship. Our evidence rule shows significant improvements in Precision and Evi-F1 value, showcasing the validity of our proposed center-sentence rule for RE. Despite the low Recall rate of our models, which may result in missing some evidence, it also helps in avoiding excessive noise introduction.

**Table 1:** Evidence extraction results on development set (%). The optimal results are shown in bold.

Model	Precision	Recall	Evi-F1
DRE-EA-path (Xu et al., 2022)	52.56	**94.20**	67.47
HESRE (Zhu et al., 2023)	57.13	92.62	70.65
Ours	**58.16**	90.72	**70.88**

Based on the above rules, we can identify the evidence more accurately, which will play an important role in the subsequent evidence graph construction and relationship prediction. By finding the center-sentence containing entity and relationship information, the extraction accuracy and completeness of evidence can be improved. The supplement of this rule helps to construct a more accurate evidence graph and better understand the relationship between entities.

### Encoding module

To enhance our ability to mine the potential semantic information of documents, we mainly employ the pre-trained BERT language model for encoding, which transforms each word in a given input document into feature vectors that represent their semantic meanings. Specifically, we then input the word ${w_i}(1 \le i \le k)$ from the document or the evidence set $D = \left[ {{w_1},{w_2}, \ldots ,{w_k}} \right]$ into BERT to obtain the embeddings.

(1) $$E = \left[ {{h_1},{h_2}, \ldots ,{h_k}} \right] = BERT(\left[ {{w_1},{w_2}, \ldots ,{w_k}} \right])$$

where *E* represents the context-embedded representation of the input word, and ${h_i}$ represents the corresponding hidden state vector generated by the $i$-th word in the input sequence.

### Graph construction and representation module

#### Evidence graph construction

To reduce the noise interference of irrelevant sentences, inspired by Liu et al. (2023), we propose to integrate the evidence into the graph, and connect the mention pairs with the same evidence in the evidence set to mine the potential semantic information between the mention pairs under the same evidence.

To simplify the structure of the evidence graph and reduce the complexity of the model, our evidence graph consists of three different types of nodes. The mention node is obtained by using the attention mechanism on the weighted representation of the words constituting the mention. The evidence corresponding to a mention pair is treated as an evidence node. The pseudo-document node is a collection of evidence for all pairs of mentions in the document.

Connecting the constructed nodes to each other with edges, we construct three different categories of edges in the evidence graph.

- Intra-entity edge: To better represent the semantic information within an entity, we establish intra-entity edges to connect different mentions to the same entity.
- Evidence edge: Different mention pairs with the same evidence are connected through evidence edge. It can effectively capture the correlation between different evidence and reduce the repeated occurrence of the same information in the model. Entities and facts involved in different evidence are complex and diverse. By establishing side connections between the same evidence, the model can better understand and infer the relationship between entities and facts, and dig deeper connections between entities. Build a global information framework to collect more path information and provide more available information for subsequent tasks.
- Mention-pseudo document edge: Evidence for all the mention is combined together in the order of the original document to construct a pseudo document node, and nodes for all the mention in the document are linked to the pseudo document node. Compared with document nodes in Zeng et al. (2020), pseudo-document node can play the same role in long-distance dependence modeling, shorten the distance between entities across sentences, and greatly reduce the interference of irrelevant sentences on entity relationship prediction.

Then, we use the GCN to learn information from different edge types in the evidence graph. The aggregation operation is carried out on the evidence graph. The representation vector of the nodes is constantly updated according to the neighbors of the nodes in the graph, so as to capture the semantic and structural relations between the nodes in the graph. The graph convolution operation is defined as:

(2) $$h_i^{(l + 1)} = \sigma \left( {\sum\limits_{k \in \Gamma } {\sum\limits_{v \in {{\rm \mathcal{N}}_k}(i)} {W_k^{(l)}} } h_v^{(l)} + b_k^{(l)}} \right)$$

where $\sigma$ represents the activation function (*e.g*., ReLU), $\Gamma$ represent different types of edges, $W_k^{(l)}$ and $b_k^{(l)}$ are trainable parameters. $${{\cal \mathcal{N}}_k}(i)$$ represents the neighbor of the connected node $u$ in *K*-th type edge.

The different layers of GCN represent the features of the different abstraction layers, so in order to cover the features of all layers and take full advantage of the representations of each layer, we connect the hidden states of each layer to form the final representation of node $i$:

(3) $${n_i} = [h_i^{(0)};h_i^{(1)}; \ldots ;h_i^{(N)}]$$

where $h_i^{(0)}$ is the initial representation of node $i$.

#### Entity-level graph reasoning

In order to better model the contextual information between cross-sentence entities, we transform the evidence graph into entity-level graph to to further simplify the redundant nodes and edge information in the graph.

The graph consists mainly of entity nodes aggregated from different mentions of the same entity, which is represented as:

(4) $${e_i} = \log \sum\limits_{i = 1}^{T({e_i})} {\exp } ({n_i})$$

where $T({e_i})$ is the number of mentions of entity ${e_i}$ in document.

All edges are used to connect different entity nodes to simulate the information interaction between different entities. The edge structure is represented by $e = [{e_1};{e_2}; \ldots ;{e_n}]$, where [;] represent the connection.

Specifically, to facilitate modeling the correlation between different entities, we combine the references of the same entity in the evidence sentence graph into entity nodes, gather the scattered information of the reference nodes together, find all the path information between the head and tail entities. Additionally, we employ a method that utilizes the attention mechanism to assign weights to each path representation. This helps in increasing the importance of more useful edge weights, thereby distinguishing different path information between the entity pairs. The goal is to identify the path information with the shortest distance between the two entities and the largest edge weights as much as possible. Subsequently, we combine the inference path information between the two entities into the final path representation, in order to learn the potential semantic information of the entities. The input ${p_{{e_{ij}}}}$ of the inference path between entity ${e_i}$ and entity ${e_j}$ is represented as:

(5) $${p_{{e_{ij}}}} = \sigma ({w_r}[{e_i};{e_j}] + {b_r})$$

where $\sigma$ is the activation function (*e.g*., ReLU), ${w_r},{b_r}$ are trainable parameters, and [;] represent the connection. ${p_{{e_{ij}}}}$ is the input to the inference path.

GCN uses graph convolution operation to realize information transfer between nodes, help entity nodes share information in the graph structure, select the best path information from numerous path information, avoid some invalid path information, thus reducing the complexity of reasoning, shortening the distance between entities, and reducing information loss in the reasoning path. The inference path information in the entity level graph is represented as:

(6) $$s = soft{\mathrm{max}}\left({{{p_{{e_{ij}}}}} \over {\sqrt d }}\right)Nsu{m_i}$$

where $Nsu{m_i}$ represents a representation of the set of all node types that make up the $i$-th subgraph and $d$ is the dimension of ${e_i}$.

We employ the attention mechanism to calculate their attention weights, capturing the relationship between entities. The path inference information *I* between entity ${e_i}$ and entity ${e_j}$ is represented as:

(7) $$I = \sigma ({w_s} \cdot [Nsu{m_i};s])$$

where $[ \cdot ]$ represent dot product and ${w_s}$ is trainable parameter.

#### Classification module

In relation prediction, the relation between entities is predicted through the interaction information and context representation between entities. We first connect the head and tail entity representation with the inference path information between them, and learn the final entity representation between them. Then, apply the activation function to map the probability value predicted by the relation type to the range (0,1), and the relation $r$ is judged by the probability value:

(8) $$P(r|{e_i},{e_j}) = sigmoid({w_r}[{e_i};{e_j};I] + {b_r})$$

where ${w_r}$ and ${b_r}$ are adjustable parameters, [;] represent the connection, *I* represent the inference path information.

Finally, we use the binary cross-entropy loss function for parameter estimation, both $i$ and $j$ are in the range of (1, $n$).

(9) $${{\mathscr{L}}} = - \sum\limits_{D{{\in \mathbb{C}}}} {\sum\limits_{i \ne j} {\sum\limits_{r \in R} r } } \log P(r|{e_i},{e_j}) + (1 - r)\log (1 - P(r|{e_i},{e_j}))$$

where $\mathbb{C}$ is the entire *corpus* and *R* is the set of relational types.

## Experiments

### Experiment settings

#### Dataset

To evaluate the performance of the model, we conducted experiments on DocRED and RE-DocRED dataset. The data statistics of the two datasets are shown in Table 2. DocRED (Yao et al., 2019) dataset stands as one of the most extensive, manually annotated collections for document-level RE presently accessible, it is also set up for large-scale remotely supervised data. Re-DocRED (Tan et al., 2022) dataset provides a high-quality re-labeling on top of the DocRED dataset, mitigating the problem of the false negative examples present in DocRED.

**Table 2:** Statistics of data used for the two benchmark settings.

Dataset	#Train	#Dev	#Test	#Relations	Avg.#Ents per Doc.
DocRED	3,053	1,000	1,000	97	19.5
RE-DocRED	3,053	500	500	97	19.4

#### Implementation details and evaluation metrics

We utilize the BERT-base-uncased (Devlin et al., 2018) as the encoder and the AdamW (Loshchilov & Hutter, 2017) as the optimizer on both datasets. Hyperparameters are adjusted based on the development set and are constantly updated during training. The detailed experimental parameter settings are shown in Table 3.

**Table 3:** Hyperparameter setting for model training.

Dataset	Batch size	Learning rate	Epoch	Dropout	Negative *vs*. Positive
DocRED	5	0.002	200	0.5	4
RE-DocRED	4	0.001	180	0.5	4

To be fair, we follow the same setup in previous work. We evaluate for DocRED RE performance using F1 and AUC. We also use Ign F1 and Ign AUC, which calculate F1 and AUC, excluding common relational facts in training and development/testing sets. In addition, we evaluate the performance of the model using intra-sentence F1 and inter-sentence F1, where intra-sentence relationships refer to a pair of entities within a sentence and inter-sentence relationships refer to a pair of entities spanning multiple sentences. For RE-DocRED, since it nicely mitigates the problem of false negative examples in DocRED, we evaluate RE performance using F1 and Ign F1.

We compare our model to the following three baseline models:

***Sequence-based models.*** These models introduce different neural network structures to encode input documents, including convolutional neural network (CNN) (Yao et al., 2019), long short-term memory (LSTM) (Yao et al., 2019), bidirectional long short-term memory (BiLSTM) (Yao et al., 2019), Context-Aware (Yao et al., 2019), and HIN-Glove (Tang et al., 2020).

***Graph-based models.*** These models build the form of a document graph from a given entity, and then simulate the relationship extraction between entities through the relationship between nodes in the graph. Graph-based models list below:

- Edge oriented graph (EoG) (Christopoulou, Miwa & Ananiadou, 2019): A model that uses different types of nodes and edges to create a document graph. The inference mechanism at the edge of the graph enables it to use internal multi-instance learning to learn relationships within and between sentences.
- Graph enhanced dual attention (GEDA) (Li et al., 2020): A model that describes complex interactions between sentences and potential relationship instances through a graph-enhanced double attention network.
- Graph Aggregation-and-Inference Network (GAIN) (Zeng et al., 2020): The model constructs two graphs, a heterogeneous reference level graph (MG) and an entity level graph (EG). The former captures complex interactions between different mentions, while the latter aggregates potential mentions of the same entity.
- Discource aware RE model (DISCO) (Wang et al., 2021): The model selects the appropriate evidence and displays the inference process on a new document graph, indicating valid semantic associations between multiple text units.
- Reconstruction (RECON) (Xu, Chen & Zhao, 2021): A method that employs a reconstruction approach to guide the DocRE model to focus more on the learning of entity pairs with real relationships.
- GRaph information Aggregation and Cross-sentence Reasoning network (GRACR) (Liu et al., 2023): A new document-level RE model with graph information aggregation and cross-sentence inference network. The model builds a simplified document-level graph to simulate the information in the document, and designs an entity-level graph to explore the relationship between long-distance entity pairs.

***Transformer-based models.*** These models improve relation extraction performance by pre-training model BERT. Transformer-based models list below:

- Hierarchical inference network (HIN) (Tang et al., 2020): This model proposes a hierarchical inference network to make full use of the rich information at entity level, sentence level and document level, and effectively aggregate the inference information from these three different granularity.
- Latent structure refinement (LSR) (Nan et al., 2020): A new model that enhances relational reasoning across sentences by automatically generalizing potential document-level diagrams. The model proposes a refinement strategy that enables the model to incrementally aggregate relevant information.
- Structured Self-Attention Network (SSAN) (Xu et al., 2021): A model that effectively combines such a structural prior while interactively performing contextual and structural inference of entities.
- Multi-view Inference framework for relation extraction with uncertain knowledge (MIUK) (Li et al., 2021): This model designs a multi-view reasoning framework, which systematically integrates local context and global knowledge in reference view, entity view and concept view.
- Adaptive Thresholding and Localized cOntext Pooling (ATLOP) (Zhou et al., 2021): This model proposes adaptive threshold and local context pooling techniques, which help alleviate the multi-entity problem.
- SSAN+KIRE (Wang et al., 2022): Based on SSAN (Xu et al., 2021), this model adds an entity knowledge injection framework to enhance document-level RE.

### Experiment results

Tables 4 and 5 present the outcomes of experiments on the two datasets. Compared to the GRACR and GAIN models, which are the most relevant for our approach, we observe better performance gains, mainly due to the fact that our model excludes the influence of irrelevant redundant sentences in the document, thus reducing the interference of noise in relation prediction.

**Table 4:** Results on the DocRED dataset. Some results are quoted from relevant paper. The results with an asterisk (*) denote those obtained in our local experiments. The optimal results are shown in bold.

Groups	Model	Dev				Test	
		Ign F1	Ign AUC	F1	AUC	Ign F1	F1
Sequence-based	CNN (Yao et al., 2019)	41.58	36.85	43.45	39.39	40.33	42.26
	LSTM (Yao et al., 2019)	48.44	46.62	50.68	49.48	47.71	50.07
	BiLSTM (Yao et al., 2019)	48.87	47.61	50.94	50.26	48.78	51.06
	Context-aware (Yao et al., 2019)	48.94	47.22	51.09	50.17	48.40	50.70
	HIN-Glove (Tang et al., 2020)	51.06	–	52.95	–	51.15	53.30
Graph-based	EOG (Christopoulou, Miwa & Ananiadou, 2019)	51.06	–	52.15	–	49.48	51.82
	GEDA (Li et al., 2020)	54.52	–	56.16	–	53.71	55.74
	GAIN* (Zeng et al., 2020)	58.19	52.48	60.28	55.69	57.72	60.13
	DISCO (Wang et al., 2021)	55.91	–	57.78	–	55.01	55.70
	RECON (Xu, Chen & Zhao, 2021)	58.13	–	60.18	–	57.12	59.45
	GRACR (Liu et al., 2023)	57.85	–	59.73	–	56.47	58.54
Transformer-based	HIN (Tang et al., 2020)	54.29	–	56.31	–	53.70	55.60
	LSR (Nan et al., 2020)	52.43	–	59.00	–	56.97	59.05
	SSAN (Xu et al., 2021)	57.03	–	59.19	–	55.84	58.16
	MIUK (Li et al., 2021)	58.27	–	60.11	–	58.05	59.99
	ATLOP* (Zhou et al., 2021)	**58.75**	–	60.66	–	**58.32**	**60.43**
	SSAN + KIRE (Wang et al., 2022)	57.29	–	59.31	–	56.31	58.65
Ours	ECRG	58.60	**55.22**	**60.73**	**58.32**	57.74	60.16

**Table 5:** Results on the RE-DocRED dataset. Some results are quoted from relevant article. The results with * denote those obtained in our local experiments. The optimal results are shown in bold.

Model	Dev		Test	
	Ign F1	F1	Ign F1	F1
GAIN* (Zeng et al., 2020)	73.96	75.22	73.85	75.12
JEREX (Eberts & Ulges, 2021)	69.12	70.33	68.97	70.25
ATLOP (Zhou et al., 2021)	73.62	74.34	73.53	74.23
SSAN (Xu et al., 2021)	72.59	74.01	71.95	73.37
SSAN + PRiSM (Choi, Lim & Choo, 2023)	73.22	74.65	72.37	73.80
ECRG	**74.17**	**75.38**	**74.10**	**75.34**

Specifically, on the DocRED dataset, it was observed that our model generally outperformed the baseline model approach on both the development and test sets. Compared with the graph-based method, we have a significant improvement in all evaluation indicators. Compared with GRACR, we have an improvement of 1.00% and 0.75% on F1 and Ign F1 in the development set and 1.62% and 1.27% on F1 and Ign F1 in the test set, respectively. Compared with GAIN, we have an improvement of 0.45% and 0.41% on F1 and Ign F1 in the development set, respectively. Compared with ATLOP, we have an improvement of 0.07% on F1 in the development set. Compared with most baseline models, the ECRG model has a clear advantage, which indicates the importance of using high-quality evidence sentence composition to mine deep semantic information, but the performance of the ECRG model is not as good as that of the most advanced model ATLOP. This phenomenon shows that the local context pool technology proposed by ATLOP can indeed capture the relevant context of entity pairs, thus alleviating the multi-entity problem and improving the performance of relation extraction. However, on the higher quality dataset of RE-DocRED, ECRG outperforms ATLOP. Our analysis believes that compared with DocRED, RE-DocRED has more standardized data and higher data quality, covering the diversity and complexity required by the task, and can fully represent the real application scenario, which can play a more fair effect in evaluating the quality of the model. Therefore, the ECRG model is more effective on RE-DocRED than ATLOP, which shows the advantages of our model. In order to analyze the model more intuitively, the performance of the model is further investigated on the extraction of inter-sentence and intra-sentence relations.

Moreover, we believe that because ATLOP uses this end-to-end black box mechanism, it is not as interpretable as our model. In addition, the extensibility of the model is more convenient for subsequent evidence sentence extraction or embedding in other methods.

However, it is worth noting that results from the test set in DocRED significantly differed from those of the training set. We hypothesize that this discrepancy may be due to certain evidence being difficult to comprehend due to the complexity of linguistic expressions, leading to less accurate associative relations in the compositions.

On the RE-DocRED dataset, our model has a significant improvement over sequence-based and graph-based models, which greatly improves the performance of relation extraction. Compared with ATLOP, we have an improvement of 1.04% and 0.55% on F1 and Ign F1 in the development set and 1.11% and 0.57% on F1 and Ign F1 in the test set, respectively. The above experimental results further demonstrate the effectiveness of our methods.

**Figure 4:**
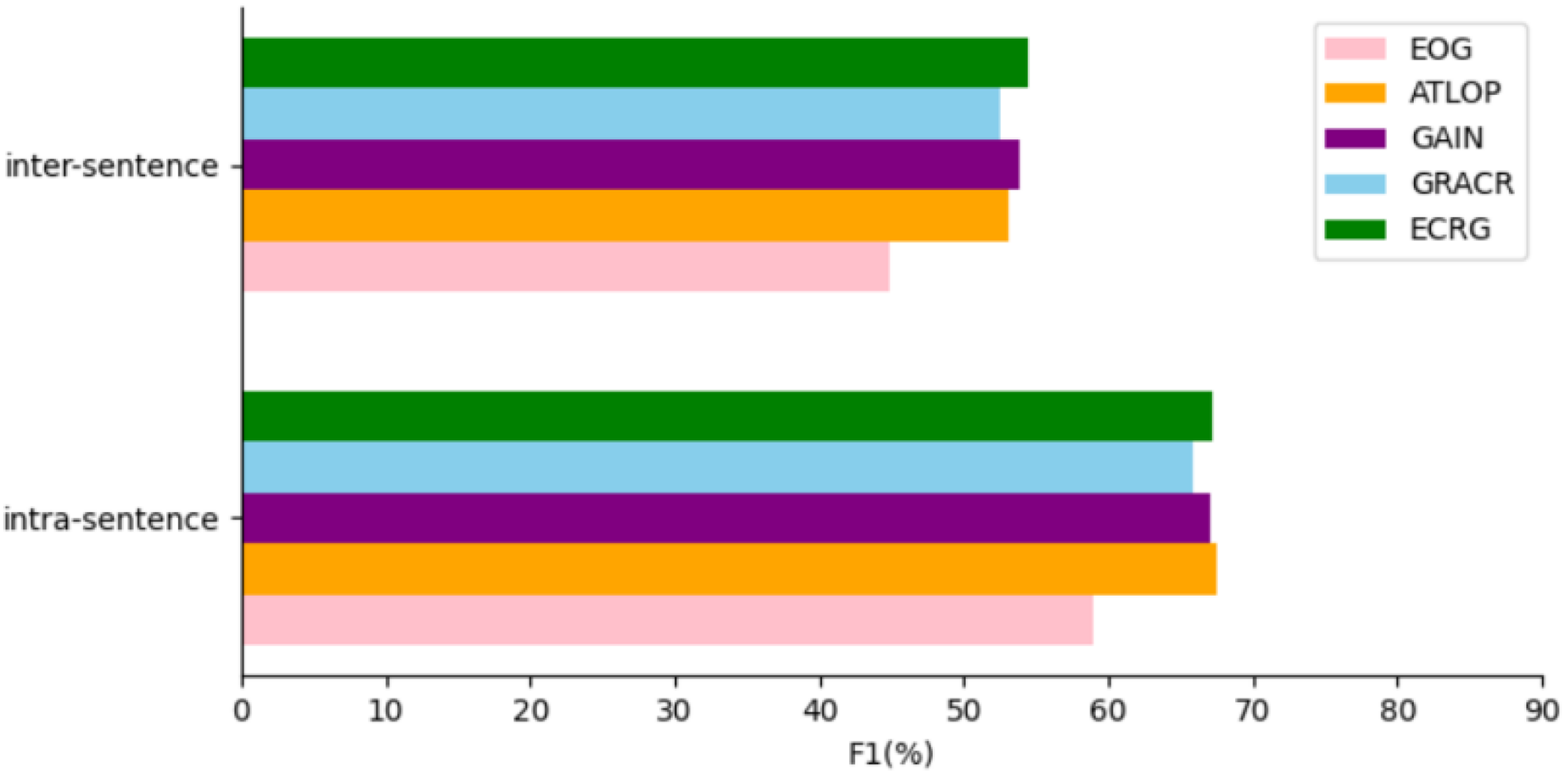
Comparison of ECRG model and other models performance Intra-F1 and Inter-F1 on the DocRED development dataset.

In order to better evaluate the performance of our model in the relation extraction of cross-sentence, we do an experimental analysis of the model in the intra-sentence and inter-sentence relation extraction of the DocRED dataset. The experimental results are shown in Fig. 4. In the intra-sentence relation extraction performance, ECRG is second only to ATLOP in relation extraction performance, and exceeds other model baselines. We think that the main reason why F1 value in sentences is lower than ATLOP is that ATLOP’s local context pool technology plays a key role. The overall performance of ECRG intra-sentence relation extraction is excellent. In the inter-sentence relation extraction performance, ECRG has more advantages than other models in the performance. Our analysis is mainly because ECRG uses efficient evidence sentences to map out more effective inter-sentence semantic information, which is helpful to improve the performance of inter-sentence relationship extraction. The experiment on the extraction of intra-sentence and inter-sentence relationships reflects that the model improves the extraction performance of intra-sentence and inter-sentence relationships. The experimental results also verify the effectiveness of the evidence extraction module and evidence graph module in ECRG in solving intra-sentence and inter-sentence relationships, especially for the semantic information capture between sentences.

### Ablation study

To demonstrate the efficacy of each individual component within the model, we conducted thorough ablation experiments on the DocRED dataset on the important components of the model, and the results present in Table 6.

**Table 6:** Ablation study results on the development set of the DocRED dataset. The optimal results are shown in bold.

Model	Dev				Test	
	Ign F1	Ign AUC	F1	AUC	Ign F1	F1
ALL	**58.60**	**55.22**	**60.73**	**58.32**	**57.74**	**60.16**
w/o evidence graph	57.86	54.49	60.00	57.71	57.24	59.61
default rules	58.09	53.77	60.09	56.85	57.28	60.09
w/o center-sentence rule	58.10	53.62	60.19	56.75	57.29	59.52
w/o evidence edge	58.15	53.83	60.23	57.07	57.71	59.92
w/o mention-pseudo edge	58.09	53.38	60.03	56.56	57.68	59.81
w/o Intra-entity edge	58.08	53.60	60.19	56.74	57.61	59.95

**Figure 5:**
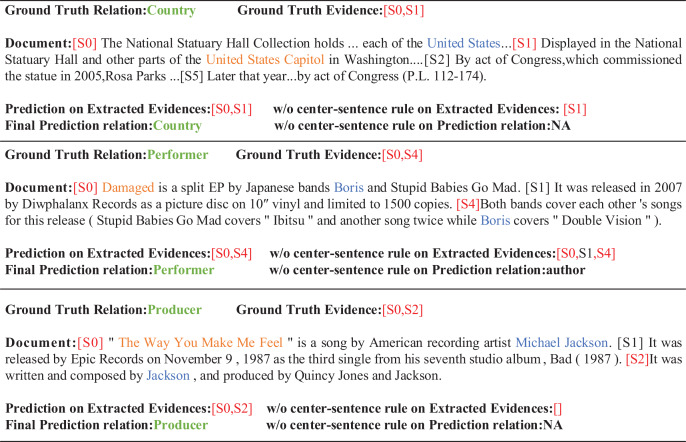
Case studies of our proposed framework ECRG. We use orange, blue, green and red to color head entity, tail entity, relation and evidence, respectively.

Firstly, we remove the center-sentence rule. The F1 value and Ign F1 value in the development set decreased by 0.54% and 0.5%, and the F1 value and Ign F1 value in the test set decreased by 0.64% and 0.45%, respectively. The ablation experiment further shows that the center-sentence rule proposed by us plays an important role in evidence extraction and relationship prediction. It is pointed out that the removal of the central sentence rule will reduce the efficiency of evidence sentence extraction, resulting in poor quality of evidence sentences, and introduce more useless and noisy sentences, which will eventually lead to the decline of the performance of relation extraction. Our analysis indicates that the center-sentence rule contains crucial contextual information about the entity. If this information is removed, the model is unable to obtain the necessary information for constructing relations completely. For example, in the case studies shown in Fig. 5, when the center-sentence rule is removed, the extraction accuracy of evidence sentences is biased to different degrees, resulting in the extraction error of evidence sentences. Then we replace the center-sentence rule with the default rule, and compared with our model performance, F1 value and Ign F1 value of the development set decreased by 0.64% and 0.51%, and F1 value and Ign F1 value of the test set decreased by 0.07% and 0.46%, respectively. We believe that the original default rule is not very effective, probably due to its insufficient consideration of realistic document context information. In summary, two ablation experiments further demonstrate the validity of the center-sentence rule.

Then, we remove the evidence graph module and found that the F1 values of the development set decrease most significantly. This may be due to the fact that the evidence graph helps the model to better synthesize the relevant information different sentences, and captures the semantic associations of documents more comprehensively. When this module is removed, the model’s understanding of the semantics between sentences is reduced, which may cause semantic ambiguity. This experiment further verifies that the evidence graph plays an important role in the model performance.

Finally, we conduct a thorough ablation experiment on the development set of each component of the evidence sentence graph to further analyze the effect of each edge on the entity relationship prediction. After removing intra-entity edges, evidence edges, and mention-pseudo document edges, respectively, the model performance decreases in each metric to varying degrees, which further suggests that all of our edge components in the evidence graph positively affect the model performance.

### Case studies

Figure 5 shows several examples of our proposed model ECRG. In the first example, the head entity is in sentence 0 and the tail entity is in sentence 1. We can see that ECRG correctly extracts the relationship *Country* between the entities *United States* and *United States Capitol*, and correctly extracts the set of evidence as [S0,S1]. After removing the center-sentence rule, the extracted evidence is only [S1]. It may be because the original default rules lack the discrimination of evidence between some entity pairs, and the predicted relationship between entity pairs is NA. This reflects the importance of the center-sentence rule for the extraction of evidence. For some cases where it is not easy to use rules to get evidence, it is often more reliable to use the center-sentence rule, because the sentence 0 contains more entity types and is more likely to be evidence. In the second example, the evidence obtained without using the center-sentence rule are [S0,S1,S4], and the relationship obtained is *author*. For the head entity *Boris* and the tail entity *Damaged*, the actual evidence are [S0,S4]. The fact relationship was *Performer* to show the importance of the center-sentence rule for evidence extraction and the importance of the evidence graph constructed after adding the rule for entity relationship prediction. In the final example, the prediction of the relationship between the evidence and the entity is correct after adding the center-sentence rule. The above example further demonstrates the importance of our proposed topic sentence rule and the use of evidence graphs for RE.

## Conclusion

In this article, we propose an enhancing cross-evidence reasoning graph for document-level RE. Specifically, first of all, we add an evidence extraction rule based on the center-sentence to extract high-quality evidence and model them as pseudo document. In addition, we use this rule to construct an evidence graph, capture the interaction information between the entity pairs with evidence in the document, and dig deep into the relationship between the entities with the same evidence. Then, based on the evidence graph, we construct an entity-level graph to capture the interaction of remote entity pairs and predict the relationship between the entity pairs. Our model shows excellent performance on both datasets and significantly improves the performance of relationship prediction between entity pairs. In the future work, we will further study the incomplete or inaccurate structure of the constructed graph due to the failure of the model to capture some important key information or concepts, and focus on improving the model’s ability to understand the semantic information of the evidence.

## Supplemental Information

10.7717/peerj-cs.2123/supp-1 Supplemental Information 1 Experimental source code describes the experimental process.

## Additional Information and Declarations

### Data Availability

The following information was supplied regarding data availability: The datasets and codes are available at GitHub: - https://GitHub.com/thunlp/DocRED - https://GitHub.com/tonytan48/Re-DocRED The code is available in the Supplemental File.
